# Vogt-Koyanagi-Harada syndrome and Crohn’s disease: an exceptional association

**DOI:** 10.1093/gastro/gov056

**Published:** 2015-11-02

**Authors:** Ahlem Souguir, Aya Hammami, Wafa Dahmeni, Hanene Jaziri, Imed Ben Mansour, Ahlem Zayene, Aida Ben Slama, Mehdi Ksiaa, Ahlem Brahem, Salem Ajmi, Ali Jmaa

**Affiliations:** Department of Gastroenterology, University Hospital Sahloul, Sousse, Tunisia

**Keywords:** Vogt-Koyanagi-Harada disease, granulomatous inflammation, Crohn’s disease

## Abstract

Vogt-Koyanagi-Harada disease (VKH) is a rare, multisystem disease of melanocyte-containing organs. It is characterized by diffuse, granulomatous inflammation involving various organs. It has been reported to occur in association with other autoimmune disorders. We report the case of a female patient who was diagnosed with VKH at the age of 4 years and who was treated with corticosteroids until the age of 16. Twenty years later, Crohn’s disease was diagnosed, with a severe flare-up. Three cases of VKH associated with ulcerative colitis have previously been reported anecdotally but, to our knowledge, this is the first case occurring in association to Crohn’s disease.

## Introduction

The Vogt-Koyanagi-Harada (VKH) syndrome is an uncommon disorder characterized by uveitis, neurological and cutaneous abnormalities (tinnitus, vertigo, meningoencephalitis, vitiligo, alopecia, and poliosis). It is also associated with other autoimmune disorders, such as autoimmune polyglandular syndrome type 1, hypothyroidism, diabetes mellitus, and Hashimoto's thyroiditis [1, 2]. Three cases of VKH syndrome occurring in association with ulcerative colitis have been described in the literature [3–5]; however, as far as we know, the association of VKH syndrome and Crohn’s disease has not been reported previously. Herein we report the case of a female patient who was diagnosed with severe flare of Crohn’s disease. She had a history of Vogt-Koyanagi-Harada syndrome twenty years prior to the onset of her inflammatory bowel disease.

## Case report

**Figure 1.**
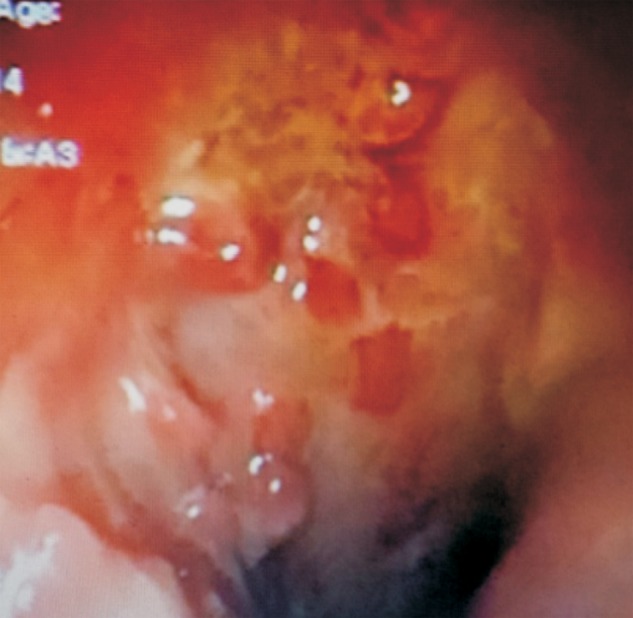
Deep ulcerations in the colon

**Figure 2.**
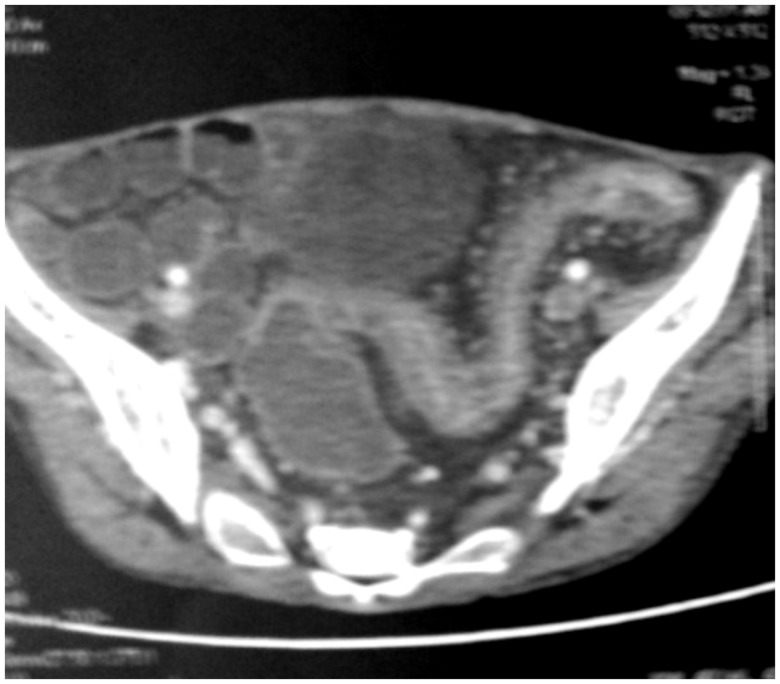
CT enteroclysis image, showing inflammatory thickening of the small intestine

A 24-year-old woman was admitted to our department with complaints of recurrent diffuse abdominal pain, with 7–10 episodes of bloodless diarrhoea and significant weight loss over the preceding 6 weeks. In her previous medical history, she had been diagnosed with VKH syndrome at the age of 4 years. The diagnosis was based on the association of bilateral granulomatous uveitis, bilateral hearing loss, cerebrospinal fluid pleocytosis and peripheral facial palsy. She was treated with corticotherapy up to the age of 16. Her follow-up revealed complete resolution of all neurological deficits except bilateral hearing loss. On physical examination, the patient looked cachectic (Body mass index: 15.3 kg/m^2^). Her vital signs were as follows: body temperature 37.2°C; blood pressure 100/50 mm Hg; heart rate 92 beats/min. Her abdominal examination showed no abnormalities. Her left lower extremity was swollen and tender, with a positive Homans sign. Laboratory investigations revealed anaemia (haemoglobin 7.3 g/dL, thrombocytosis (platelet count 953 000 cells/L), hypoalbuminaemia (18 g/L) and high C-reactive protein level (85 mg/L). On colonoscopy, we observed diffusely inflamed mucosa with circumferential deep ulcerations in the ileum and the left-sided colon (Figure 1). The diagnosis of severe flare-up was upheld. Upper gastrointestinal endoscopy revealed a simple gastritis with a normal aspect of the duodenum; however, a histological report confirmed the diagnosis of Crohn's disease involving the duodenum and the colon. A small bowel enema showed an inflammatory thickening of the small intestine (Figure 2). Doppler ultrasound of the left leg confirmed the diagnosis of a deep venous thrombosis, extending to the common iliac vein.

The patient was treated with a high dose of intravenous hydrocortisone, enteral nutrition and low-molecular-weight heparin at curative dose. Her clinical and biological outcomes were favourable at first; however, she described recurrence of abdominal pain on day 7 of corticotherapy. Consequently, an endoscopic control was performed and it showed persistence of severe lesions. Anti-tumour necrosis factor (TNF) therapy (infliximab)—at a dose of 5 mg/kg at weeks 0, 2, and 6—was then initiated to induce remission. The patient remained asymptomatic, with normal biological tests. She was thus maintained on regular infliximab infusions every 8 weeks. After two years of follow-up, her endoscopic control showed mucosal healing. No symptoms related to VKH syndrome were noticed during this period.

## Discussion

VKH syndrome (or uveomeningoencephalitis), a well-established multiorgan disorder affecting pigmented structures, is an autoimmune disorder of melanocyte proteins in genetically susceptible individuals [6]. It may affect the eyes, hair, central nervous system, inner ear, and skin [7]. VKH syndrome is more common in Asians, Native Americans, Hispanics, and Middle Easterners [8], with a female preponderance in North American patients [1]. Peak incidence occurs in patients in their thirties. Although several theories exist, the aetiology of VKH still unknown. Experimental evidence supports an autoimmune cause for VKH syndrome, since antibodies to melanin have been described in all areas of involvement [2, 9], which explains the association of this syndrome with other diseases of immune dysregulation.

Three cases of concurrent VKH and ulcerative colitis have been reported in the past [3–5]. In one, the ulcerative colitis was quiescent at the time of ocular disease activity. In the other two, patients developed severe flare of their disease, requiring colectomy in one case and treatment with anti-TNF agents in the other. Interestingly, our patient represents the first case diagnosed with both VKH syndrome and severe Crohn’s disease, which may or may not be an incidental association. We believe that immunological mechanisms probably play an important role in this association.

The concomitant association of VKH syndrome and Crohn’s disease in our patient may be accidental; however, some authors have suggested a probable pathogenic link with dysregulation of the immune system. Clinicians should be highly vigilant for this association—particularly in the presence of neurological deficits and ocular symptoms—since timely immunosuppressive therapy may lead to improved outcomes of ocular disorders.

*Conflict of interest statement*: none declared.
